# NEAT1 regulates microtubule stabilization via FZD3/GSK3β/P-tau pathway in SH-SY5Y cells and APP/PS1 mice

**DOI:** 10.18632/aging.104098

**Published:** 2020-11-18

**Authors:** Yiwan Zhao, Ziqiang Wang, Yunhao Mao, Bing Li, Yuanchang Zhu, Shikuan Zhang, Songmao Wang, Yuyang Jiang, Naihan Xu, Yizhen Xie, Weidong Xie, Yaou Zhang

**Affiliations:** 1 State Key Laboratory of Chemical Oncogenomics, Tsinghua Shenzhen International Graduate School, Shenzhen 518055, P.R. China; 2 School of Life Sciences, Tsinghua University, Beijing 100084, P.R. China; 3 Key Lab in Healthy Science and Technology of Shenzhen, Tsinghua Shenzhen International Graduate School, Shenzhen 518055, P.R. China; 4 Key Laboratory of Medical Reprogramming Technology, Shenzhen Second People’s Hospital, First Affiliated Hospital of Shenzhen University, Shenzhen 518035, P.R. China; 5 Open FIESTA Center, Tsinghua University, Shenzhen 518055, P.R. China; 6 State Key Laboratory of Applied Microbiology Southern China, Guangdong Provincial Key Laboratory of Microbial Culture Collection and Application, Guangdong Institute of Microbiology, Guangdong 510070, P.R. China

**Keywords:** Alzheimer’s disease, NEAT1, FZD3, H3K27Ac, metformin

## Abstract

Nuclear paraspeckles assembly transcript 1 (NEAT1) is a well-known long noncoding RNA (lncRNA) with various functions in different physiological and pathological processes. Notably, aberrant NEAT1 expression is implicated in the pathogenesis of various neurodegenerative diseases, including Alzheimer’s disease (AD). However, the molecular mechanism of NEAT1 in AD remains poorly understood. In this study, we investigated that NEAT1 regulated microtubules (MTs) polymerization via FZD3/GSK3β/p-tau pathway. Downregulation of NEAT1 inhibited Frizzled Class Receptor 3 (FZD3) transcription activity by suppressing H3K27 acetylation (H3K27Ac) at the FZD3 promoter. Our data also demonstrated that P300, an important histone acetyltransferases (HAT), recruited by NEAT1 to bind to FZD3 promoter and mediated its transcription via regulating histone acetylation. In addition, according to immunofluorescence staining of MTs, metformin, a medicine for the treatment of diabetes mellitus, rescued the reduced length of neurites detected in NEAT1 silencing cells. We suspected that metformin may play a neuroprotective role in early AD by increasing NEAT1 expression and through FZD3/GSK3β/p-tau pathway. Collectively, NEAT1 regulates microtubule stabilization via FZD3/GSK3β/P-tau pathway and influences FZD3 transcription activity in the epigenetic way.

## INTRODUCTION

Alzheimer’s disease (AD) is the leading cause of dementia among the aging population that involves complex neurodegenerative alterations. There are several hypotheses to explain the basis of AD. Among them, the cholinergic, amyloid-β (Aβ) and tau hypotheses are the most recognized doctrine [1–4]. Currently, the available therapy of enhancing the acetylcholine response is not very satisfactory, and the trials targeting Aβ in AD repeatedly failed [5]; therefore, the microtubule associated protein tau (MAPT) hypothesis has gained much attention. In healthy human neurons, tau binds to microtubules to regulate its stability; in AD brains, however, hyperphosphorylated tau is detached from microtubules and polymerized into paired helical filaments (PHFs), forming neurofibrillary tangles (NFTs), thus contributing to neuronal degeneration [6, 7]. Microtubules play essential roles in neuronal morphogenesis and intracellular transport. Abnormal tau phosphorylation results in microtubules architecture disruption, neurite retraction, axonal transport impairment, and consequently neuronal damage [8]. In addition, affected neurons in AD brain show a decreased number of synapses, implying a close correlation with cognitive impairment [9].

Nuclear enriched abundant transcript 1(NEAT1) is essential for the structure of nuclear paraspeckles, which is a type of nuclear bodies existing in mammalian nuclei to control gene expression and epigenetic events [10, 11]. The NEAT1 gene has two isoforms, NEAT1v1 (3.7 kb in length) and NEAT1v2 (23 kb in length), both providing the crucial structural framework to nucleate paraspeckle formation [12]. NEAT1 has gained much NEAT1 attention due to its critical roles in maintenance of nuclear bodies, chromatin remodeling, gene expression regulation, and tumor progression in different cancers [13, 14]. Aberrant overexpression of NEAT1 has been implicated in various types of solid tumors, such as lung cancer, oesophageal cancer, as well as colorectal cancer, in which its high levels are associated with poor prognosis [15]. Recent studies have shown that NEAT1 was also involved in neuronal loss diseases and neurodegenerative disorders, such as amyotrophic lateral sclerosis (ALS), traumatic brain injury (TBI), Huntington’s disease (HD) and Alzheimer’s disease (AD) [16–18]. Numerous studies have investigated the dysregulated NEAT1 in different brain regions of AD patients [19], AD mouse model [20] and amyloid-β (1-42) treated SH-SY5Y cells [21]. Emerging evidence has suggested that NEAT1 expression and miRNAs were correlated in AD [21]. MiR-124 was a directly target of NEAT1 and the expression of beta-secretase 1(BACE1) was the potential functional target of miR-124, suggesting that NEAT1 exerted function in AD development via regulating miR-124/BACE1 axis [20]. Our lab’s recent study accidentally found that NEAT1 reduced significantly in the early stage of AD. And the depletion of NEAT1 prevented neuroglial cell mediating Aβ clearance via modulating endocytosis-related genes [22]. These findings suggested that NEAT1 worked as a biomarker, as well as a potential pharmacological target for AD treatment.

Patients with type 2 diabetes are more likely to develop AD, suggesting the common biological mechanisms between them, including insulin resistance, disrupted glucose metabolism, Aβ formation, oxidative stress and so on [23, 24]. Metformin is an oral antidiabetic drug, which is considered as a promising drug for AD. In a study by Kickstein et al., metformin reduced tau phosphorylation in primary murine neurons *in vitro* and *in vivo* [25]. Chen et al. and his coworkers have reported that in Type 2 diabetic db/db mice, metformin rescued memory impairment, prevented neuronal apoptosis and Aβ accumulation [26]. In this report, we aimed to study the mechanism of NEAT1 in maintaining MTs stability. We show that the depletion of NEAT1 disrupted MTs structure in SH-SY5Y cells and murine neurons. Gene expression profiling from GEO datasets were used to perform GO analysis and found Wnt signaling pathway is enriched in NEAT1-associated genes. We investigate that NEAT1 regulate FZD3, receptor for Wnt proteins, via influencing histone modification of its promoter. Knockdown of NEAT1 reduced H3K27Ac level of FZD3 promoter via an association with P300. Decreased FZD3 expression results in inhibition disheveled proteins and activation of GSK-3 kinase, eventually following an increase in the amount of phosphorylated-tau(p-tau). Metformin have been implicated in regulating phosphorylation pattern of the AD-related tau protein. We have hypothesized that metformin, by activating NEAT1 and FZD3, would exhibit tau dephosphorylating potency. We show that metformin increases NEAT1 expression in SH-SY5Y cells and in the hippocampi of APP/PS1 mice, and further leads to the ascending FZD3 expression and a dephosphorylation of tau epitopes. Thus, we propose that metformin abandon tau hyper-phosphorylation and rescue MTs disruption via FZD3/GSK3β/p-tau pathway.

## RESULTS

### NEAT1 silencing induces de-polymerization of microtubules (MTs) in SH-SY5Y and primary murine neurons

**Figure 1:**
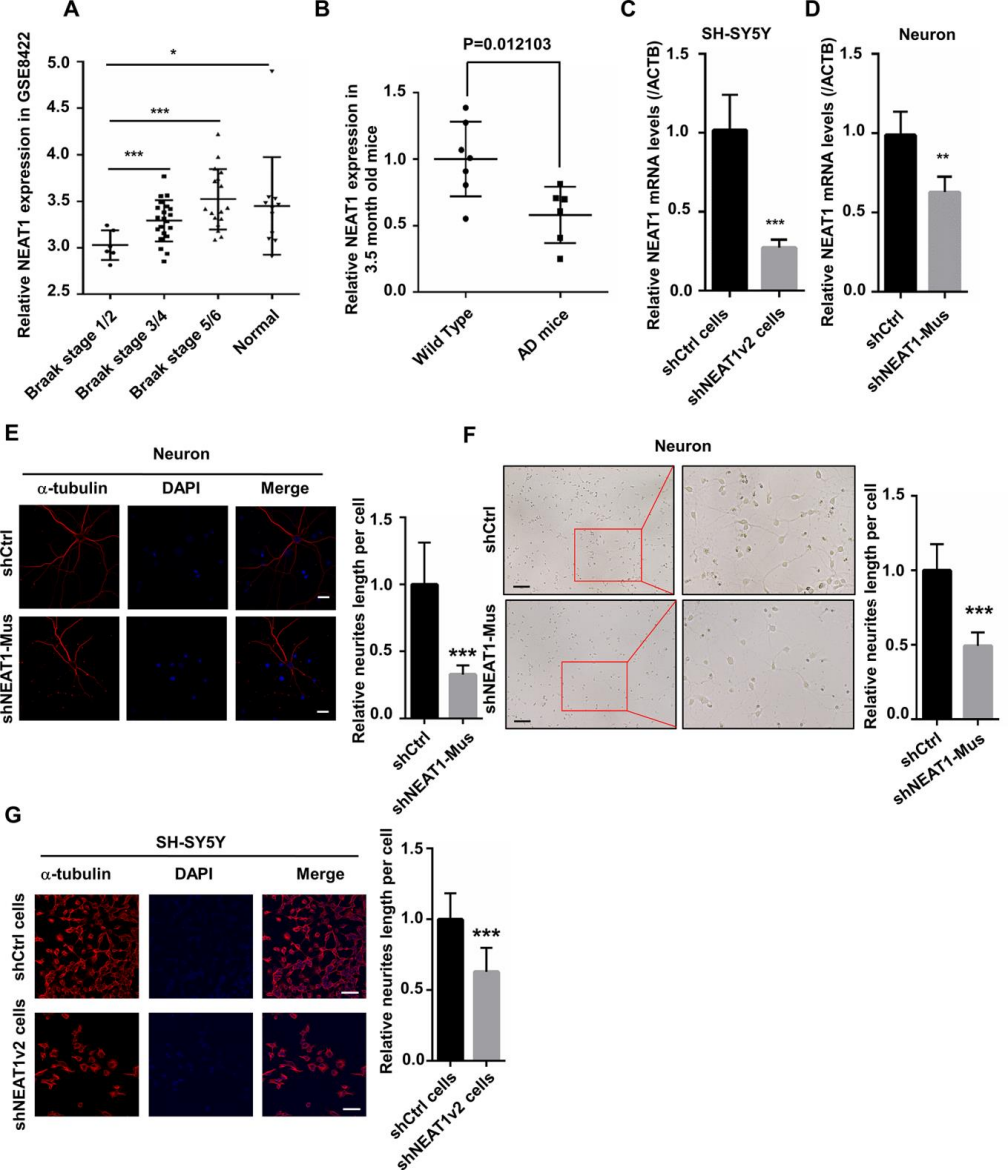
**NEAT1 silencing induces de-polymerization of microtubules (MTs).** (**A**) The expression of NEAT1 in the hippocampi of AD patients with different braak stage and normal persons was analyzed in GSE84422. (**B**) NEAT1 analysis in the hippocampi of 3.5-month-old AD mice and Wild Type. (**C**) The NEAT1 mRNA level was measured by quantitative PCR in shNEAT1v2 cells and shCtrl cells. (**D**) NEAT1 mRNA level was detected by quantitative PCR in shNEAT1-Mus and shCtrl transfected murine neurons. (**E**) Immunofluorescence staining of α-tubulin (red) in shNEAT1-Mus and shCtrl transfected murine neurons. (**F**) Morphological changes of murine neurons were observed under light microscope after NEAT1 knockdown. (**G**) Immunofluorescence analysis of α-tubulin (red) in shNEAT1v2 cells and shCtrl cells. DAPI (blue) was used to stain the nuclei. Scale bars, 20μm. Image J software was used to analyze the cell dendritic length (mean ± s.d, *P < 0.05, **P < 0.01, ***P < 0.001, Student 2-tailed *t* test).

With the help of Kolmogorov-Smirnov test, we analyzed, the expression profiles in hippocampi of different stage AD patients and normal persons using data from the National Center for Biotechnology Information (NCBI). NEAT1 expression in the hippocampi of AD patients at different stages (GSE84422) as well as normal person was shown in Figure 1A. Results showed that the expression of NEAT1 significantly reduced in Braak stage 1 and 2, representing an early-stage of AD (Figure 1A). Braak stage 1 and 2 are the earliest disease phases in AD, in which abnormal tau and neurofibrillary tangle start to appear. Interestingly in our study, quantitative RT-PCR (qPCR) revealed that NEAT1 expression in hippocampi of 3.5-month-old AD mice also decreased dramatically compared with Wild Type (Figure 1B).

To explore the mechanism of dysregulated NEAT1 during the early stage of AD, we generated NEAT1-deficient cells (shNEAT1v2 cells) and negative control cells (shCtrl cells) using lentivirus based NEAT1-targeting short hairpin RNA (shRNA) and control shRNA vectors on SH-SY5Y cells. The inhibition efficiency of SH-SY5Y cell lines is approximately up to 80% (Figure 1C). Next, murine neurons were isolated from embryonic E18.5 C57BL/6 mice and were transfected with lentivirus based shNEAT1v2-Mus and shCtrl, resulting in 50% inhibition ratio (Figure 1D). We performed immunofluorescence experiments with anti-α-tubulin antibodies, and found decreased length of neurites in NEAT1-deficient murine neurons. The images were captured using confocal microscope (Figure 1E), as well as light microscope (Figure 1F). The same phenomenon also occurred in shNEAT1v2 cells compared to shCtrl cells (Figure 1G).

**Figure 2:**
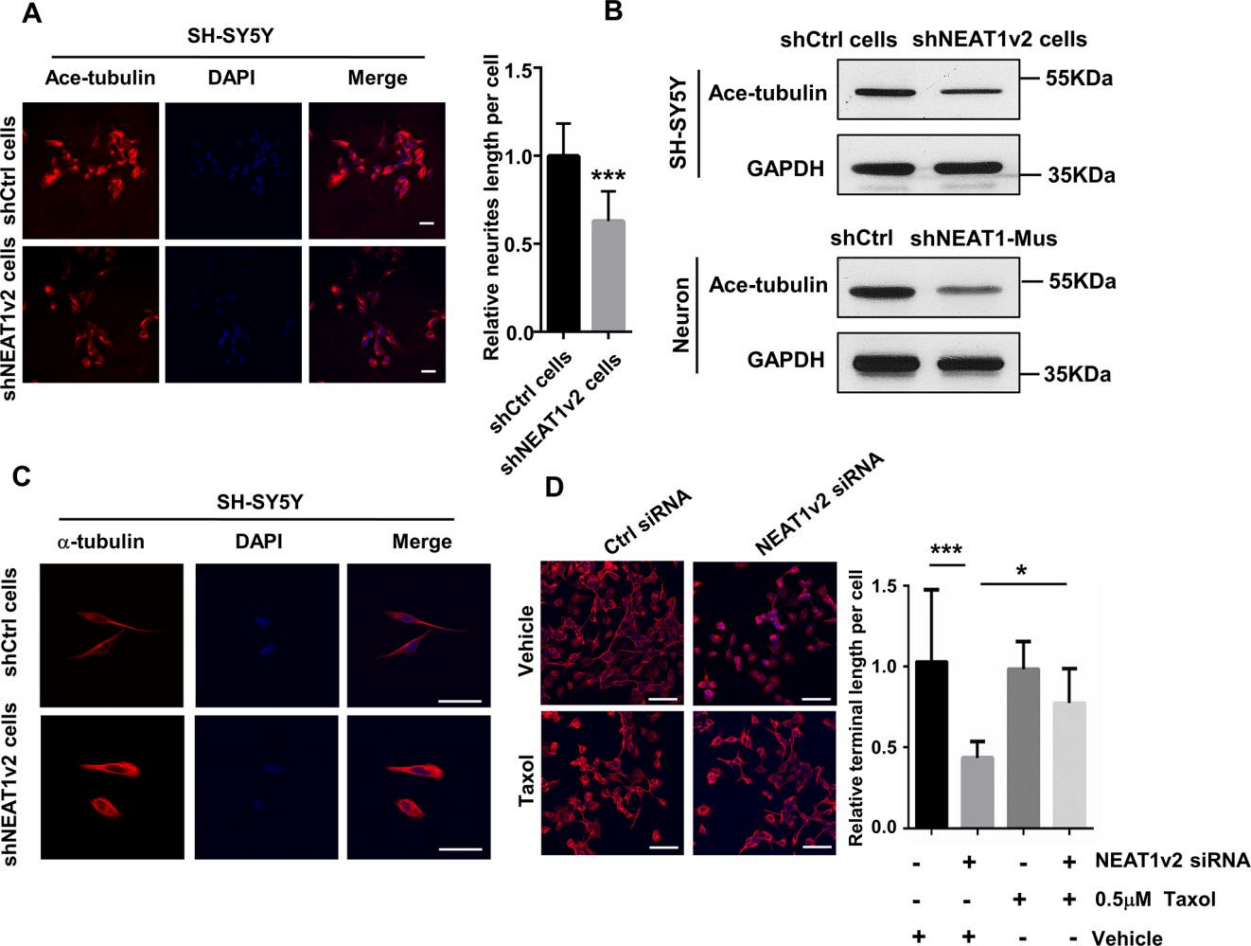
**NEAT1 silencing induces de-polymerization of microtubules (MTs).** (**A**) Immunofluorescence staining of Ace-tubulin (red) in shNEAT1v2 cells and shCtrl cells. Scale bars, 20μm. (**B**) Western blot analysis for acetylated tubulin expression in shNEAT1v2 cells and shCtrl cells as well as neurons. (**C**) Immunofluorescence analysis of α-tubulin (red) in shNEAT1v2 cells and shCtrl cells in microscope high power fields. Scale bars, 50μm. (**D**) NEAT1 siRNA and control siRNA transiently transfected SH-SY5Y cells were treated with 0.5μM taxol for 72 hours. And then immunostained with α-tubulin (red). DAPI (blue) was used to stain the nuclei. Scale bars, 100μm. Image J software was used to analyze the cell dendritic length (mean ± s.d, *P < 0.05, **P < 0.01, ***P < 0.001, Student 2-tailed *t* test).

We suggested that NEAT1 knockdown induced neurite retraction is possibly due to the de-polymerization of microtubules. We did a series of experiments to confirm our opinion. The shNEAT1v2 cells and shCtrl cells were immunostained by acetyl-tubulin antibody and results showed decreased amount of acetylated tubulin in shNEAT1v2 cells (Figure 2A). The reduced protein expression of acetyl-tubulin was also detected in shNEAT1v2 cells and NEAT1 knockdown neurons (Figure 2B). Post-translational modifications of MTs, such as acetylation and tyrosination have been identified as markers for stabilized MTs. These data indicated that NEAT1 knockdown resulted in neurites retraction and depolymerization of MTs. Besides, we observed the cell morphology of shNEAT1v2 cells in confocal microscope high-power fields, and clearly found the loose microtubules and decreased tubulin assembly (Figure 2C). Taxol drastically affects the assembly of microtubules, which is known as an agent that stabilizes microtubules. NEAT1 siRNA transfected cells were immunostained with anti-α-tubulin antibodies and we found more stable microtubules and high cell integrity in NEAT1 knockdown cells after treating 0.5μM taxol compared with the ddH2O treated group (Figure 2D).

### NEAT1 modulates MTs stability via FZD3/GSK3β/p-tau signaling pathway

**Figure 3:**
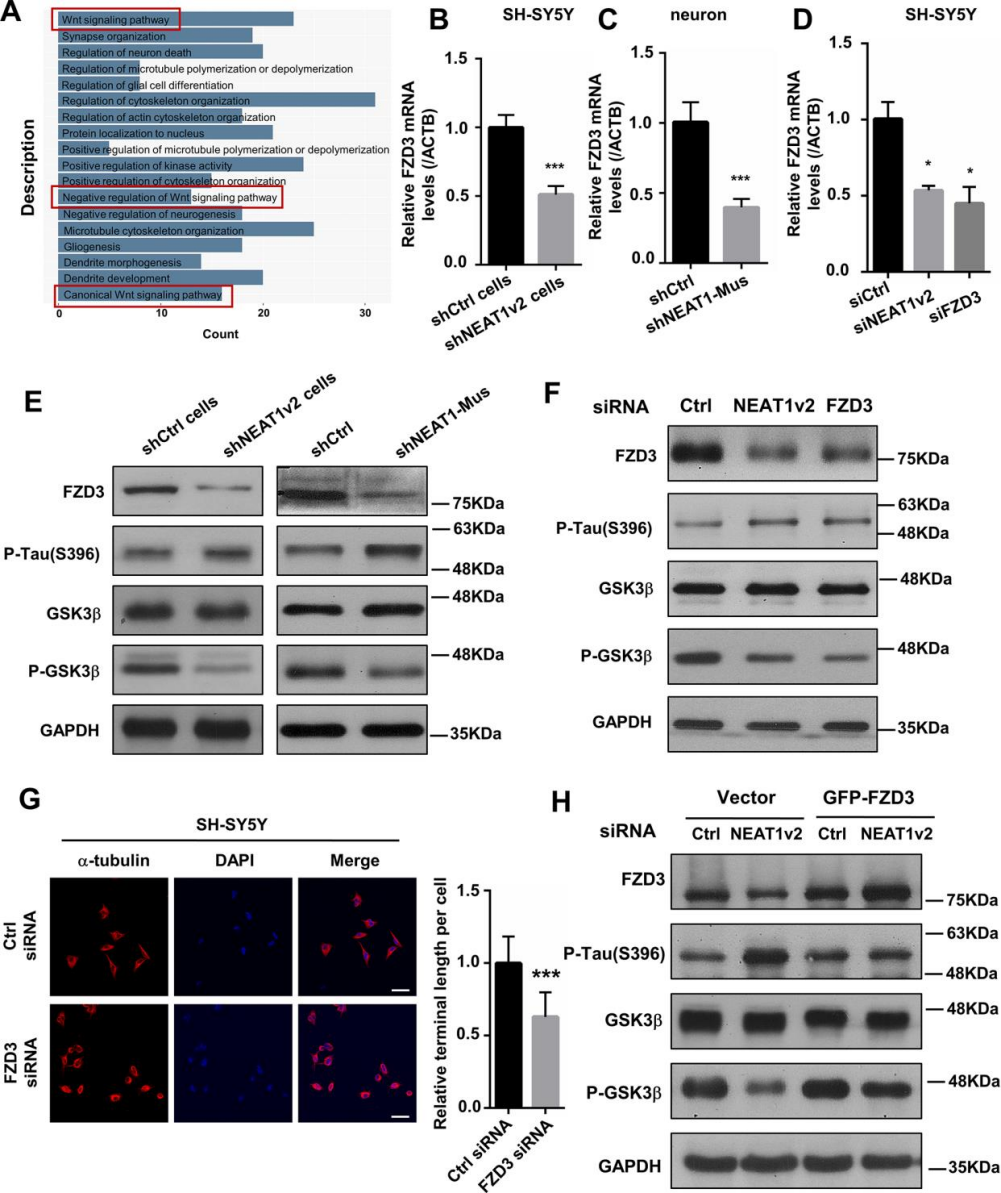
**NEAT1 silencing mediates de-polymerization of MTs via FZD3/GSK3β/p-tau signaling pathway.** (**A**) GO analyses were performed using the NEAT1-associated genes obtained from expression profile of braak stage 1/2 AD patients in GSE84422. (**B**) The FZD3 mRNA level was measured by quantitative PCR in shNEAT1v2 cells and shCtrl cells. (**C**) The mRNA level of FZD3 in shNEAT1-Mus and shCtrl transfected murine neurons. (**D**) The mRNA level of FZD3 in SH-SY5Y after being transfected with NEAT1 siRNA, FZD3 siRNA and Ctrl siRNA. (**E**) The expression levels of the FZD3, GSK3β, p-GSK3β, p-Tau(s396), Ace-tubulin and GAPDH were analyzed with immunoblotting in shNEAT1v2 cells, shCtrl cells and shNEAT1-Mus, shCtrl transfected murine neurons, respectively. (**F**) The expression levels of the FZD3, GSK3β, p-GSK3β, p-Tau(s396), Ace-tubulin and GAPDH were analyzed with immunoblotting in NEAT1v2 siRNA, FZD3 siRNA transiently transfected SH-SY5Y. (**G**) Immunofluorescence staining of α-tubulin (red) in FZD3 siRNA transiently transfected SH-SY5Y. DAPI (blue) was used to stain the nuclei. Scale bars, 20μm. Image J software was used to analyze the cell dendritic length. (**H**) The expression levels of the FZD3, GSK3β, p-GSK3β, p-Tau(s396) and GAPDH were analyzed with immunoblotting in GFP-FZD3 vector and control vector, while transfected NEAT1 siRNA (mean ± s.d, *P < 0.05, **P < 0.01, ***P < 0.001, Student 2-tailed *t* test).

To investigate the function of NEAT1 in MTs stability, a correlation analysis was performed by using data from GEO datasets (GSE84422). A cluster (|r| > 0.4) of NEAT1-associated genes obtained from expression profile of braak stage 1/2 AD patients were subjected to enrichment analysis of GO functions and KEGG pathways. GO (gene ontology) analysis suggested that NEAT1-associated genes were primarily enriched in Wnt signaling pathway (Figure 3A). We detected the expression of some important components in Wnt signaling pathway in shNEAT1v2 cells and shCtrl cells. And found several of them significantly changed, including FZD3. FZD3 involved in the development of the central nervous system, including structure plasticity and synaptogenesis [27]. Decreased FZD3 mRNA levels were determined in shNEAT1v2 cells and shNEAT1-Mus transfected murine neurons (Figure 3B, 3C). After that, we found that NEAT1v2 siRNA similarly to FZD3 siRNA down-regulated FZD3 in SH-SY5Y as well (Figure 3D).

In Wnt signaling pathway, a Wnt ligand binds to a membrane-bound complex consisting of Fz receptor family and a LDL receptor-related protein [28]. This leads to the activation of disheveled (Dvl) proteins, then accompanied by an inhibition of Glycogen synthase kinase 3β (GSK3β) [29]. GSK3β is a specific and well-studied tau protein kinase [4, 30, 31]. Immunoblotting revealed that FZD3, P-GSK3β, Ace-tubulin were significantly decreased in shNEAT1v2 cells and shNEAT1-Mus transfected murine neurons, while p-tau increased dramatically (Figure 3E). The same results were also observed in NEAT1 siRNA, FZD3 siRNA transfected SH-SY5Y cells (Figure 3F). Immunofluorescence staining demonstrated that neurites retraction and MTs de-polymerization was also observed in FZD3 siRNA transfected SH-SY5Y (Figure 3G). The reduced p-GSK3β (ser9) with unchanged total GSK3β suggested a decreased in p-GSK3β/t-GSK3β ratio (i.e., increased GSK3β activity). Phosphorylation of GSK3β at serine 9 (p-GSK3β) is a modification process required to inhibits GSK3β activity [4, 30]. The decreased in p-GSK3β /t-GSK3β ratio thereby increased an active form of GSK3β, and ultimately resulted an elevated level of Phosphorylated form of Tau protein. In order to further prove the important role of FZD3 in this pathway, we build a GFP-FZD3 plasmid to overexpress FZD3 and an empty vector as control. The results showed that FZD3 overexpression increased p-GSK3β and reduced p-tau even if knocking down NEAT1v2, suggesting NEAT1 dose regulate p-tau through FZD3 (Figure 3H).

### NEAT1 regulates FZD3 expression via interaction with histone acetyltransferase P300

**Figure 4:**
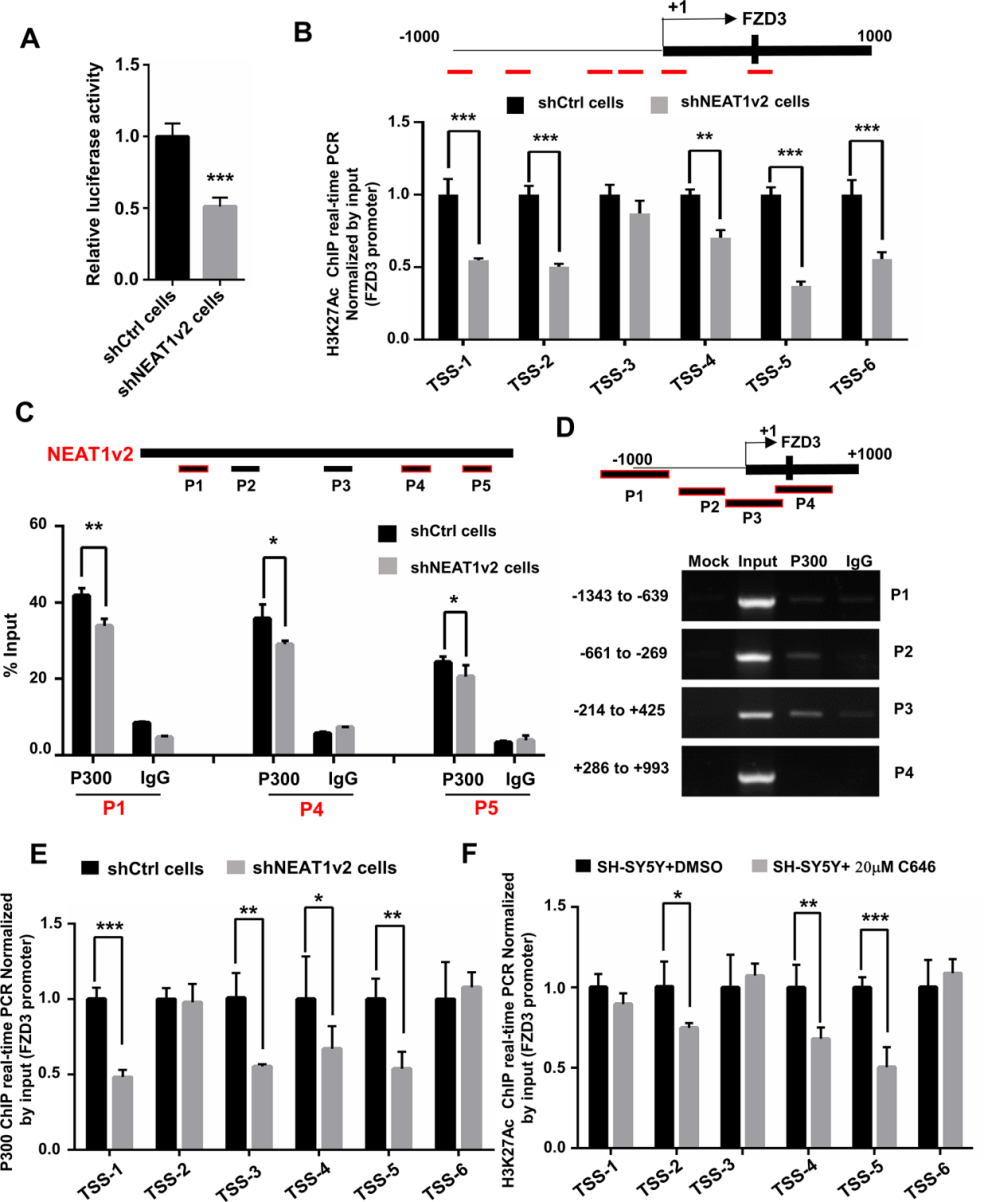
**NEAT1 regulates FZD3 expression via recruitment of histone acetyltransferase P300.** (**A**) After co-transfection with NEAT1v2 siRNA or Ctrl siRNA and the pGL3 enhancer plasmid containing FZD3 promoter fragments, the relative transcriptional activities were determined with a luciferase assay in three independent experiments. (**B**) The shNEAT1v2 cells and shCtrl cells were collected for ChIP assays to analyze the relative fold enrichment of the FZD3 promoter using anti-H3K27Ac antibody (n=3). (**C**) Schematic of potential P300 binding sites in the NEAT1 sequence. The shNEAT1v2 cells and shCtrl cells lysates were harvested and subjected to a RIP assay. QRT-PCR was performed to detect the retrieval of NEAT1 and ACTB by the anti-P300 and anti-IgG antibodies over the input level (n=3). (**D**) ChIP-PCR analysis was performed to detect the potential P300 binding sites in the FZD3 promoter after using SH-SY5Y cells lysates (n=3). (**E**) The shNEAT1v2 cells and shCtrl cells were collected for ChIP assays to analyze the relative fold enrichment of the FZD3 promoter using anti-P300 antibody (n=3). (**F**) The 20μM C646 treated and vehicle treated SH-SY5Y cells were collected for ChIP assays to analyze H3K27Ac enrichment level of the FZD3 promoter (n=3) (mean ± s.d, *P < 0.05, **P < 0.01, Student 2-tailed *t* test).

Researchers reported that NEAT1 functioned as a transcriptional regulator to mediate gene expression [32, 33]. To explore the mechanism of how NEAT1 regulate FZD3, we generated luciferase reporter constructs containing the promoter region of FZD3 and co-transfected with NEAT1v2 siRNA or Ctrl siRNA in SH-SY5Y. The results showed NEAT1 knockdown reduced the transcriptional activity of FZD3 promoter, indicating that NEAT1 regulate the expression of FZD3 at transcriptional level (Figure 4A). After that, we examined the H3K27 acetylation (H3K27Ac) status, an active transcription marker, at FZD3 promoter in shNEAT1v2 cells and shCtrl cells. To identify these modified histone H3-binding sites within the FZD3 promoter sequences, we designed sets of primer pairs (Table 1) that recognized the TSS regions of FZD3. The chromatin immunoprecipitation (ChIP) assays revealed a broad and significantly decreased enrichment of H3K27Ac at the FZD3 promoter (Figure 4B).

**Table 1:** List of primers used for ChIP analyses.

**Primers pairs**	**Sence sequence**	**Anti-sence sequence**
FZD3-Tss-1	TCAGGGATCGTTCCTCTCGT	GGCGGACAGGGTTAACAGTC
FZD3-Tss-2	TGTTTCGCGTGGAGCTCTG	TGTGATTGCAGGACCACCTAC
FZD3-Tss-3	ACCTCCCGATGTTGAGCTAT	CTCTGGAGATGTGCTGCGAG
FZD3-Tss-4	GTGTAGGTGGTCCTGCAATCA	CCTGGAGGCGCTCATCTG
FZD3-Tss-5	CCTATTCTGTCCGCTACGCT	AGGTGTGATTGCTACGCT
FZD3-Tss-6	CCGGGAGACTGTTAACCCTG	CCCCCGGAGCATTGTCTT
FZD3-Tss-P1	CATTCCCACCTCCCGATGTT	GAACGCCCCCAAAGGTTAGA
FZD3-Tss-P2	CAGGGATCGTTCCTCTCGTC	GCCAAGAAAAGCACCCTTGG
FZD3-Tss-P3	ATCTCAGATGAGCGCCTCCA	GAGGGGAAACTTTCAGGCGT
FZD3-Tss-P4	TCATCTAACCTTTGGGGGCG	GTGTGATTGCAGGACCACCT

CREB-binding protein (CBP)/P300 are transcriptional coactivators with histone acetyltransferase activity, which acetylate lysine residues on histones to modulate chromatin structure or function. And P300/CBP are absolutely essential for H3K27Ac [34]. Our previous study showed that NEAT1 recognizes and co-localizes with P300/CBP, indicating that NEAT1 affects the acyltransferase activities of P300/CBP by direct interaction with P300 [22]. To determine the interaction between NEAT1 and P300, we performed an RNA immunoprecipitation (RIP) assay using the P300 antibody and IgG antibody followed by qRT-PCR in shNEAT1v2 cells and shCtrl cells. We designed five pairs of qPCR primers that recognized NEAT1 fragments (P1, P2, P3, P4, P5) (Table 2). Results showed that NEAT1 can interact with P300 on P1, P4 and P5 regions, and recruited less P300 in shNEAT1v2 cells compared with shCtrl cells. The data suggests that the interaction of NEAT1 and P300 may regulate epigenetically FZD3 gene transcription (Figure 4C).

**Table 2:** List of primers against to NEAT1 used for RIP analyses.

**Primers pairs**	**Sence sequence**	**Anti-sence sequence**
P1	GCCTTCTTGTGCGTTTCTCG	TCCCAGCGTTTAGCACAACA
P2	TCTCAGAACCCACCTCCTGT	TCAGGGACAAGCAACAACCA
P3	GCTTAATGCTGACAAGGCCC	TGCAGGCATAAGCAGAGGAC
P4	TCTCCTGGCTATTCCAGGCT	GCCGAGGTAGACAGACCAAG
P5	CAGTCTTGCTCTAGCCCCAC	GATGGCATCAGTAGCCTCCC

Next we investigated whether P300 influence H3K27Ac level on FZD3 promoter. Firstly, we designed four paired primers (P1, P2, P3, P4) (Table 1) across the promoter region (-1000bp to +1000bp) of FZD3 and then subjected to ChIP-PCR assay to identify its regulatory mechanism. The results showed that P300 directly binds to P2 (-661bp to -269bp) and P3 (-214bp to +425bp) regions of FZD3 promoter (Figure 4D). The ChIP assay demonstrated that knockdown NEAT1 decrease the enrichment of P300 in the FZD3 promoter (Figure 4E). SH-SY5Y cells were treated with 20μM C646, a selective inhibitor of P300, for 60h and a significant reduction of H3K27Ac enrichment was observed at FZD3 promoter (Figure 4F). Taken together, our results suggested that NEAT1 recognizes and recruits the histone acetyltransferase P300 to FZD3 promoter and regulates FZD3 transcriptional activity by altering the H3K27Ac status of FZD3 promoter.

### Metformin alleviates the depolymerization of MTs and neurites retraction by increasing NEAT1

**Figure 5:**
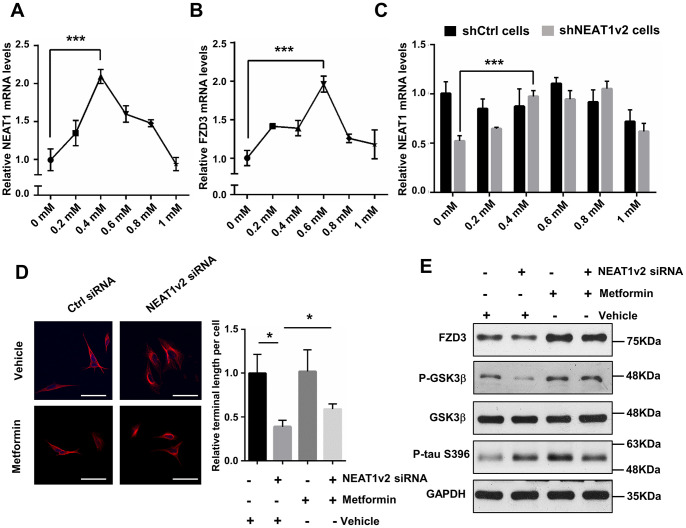
**Metformin alleviates the depolymerization of MTs and neurites retraction by increasing NEAT1.** (**A**, **B**) Quantitative PCR analysis of NEAT1 and FZD3 expression of 0 to 1mM metformin treatment on SH-SY5Y cells for 48h. (**C**) The NEAT1 expression was detected in shNEAT1v2 cells and shCtrl cells after treated with 0 to 1mM metformin. (**D**) The NEAT1v2 siRNA and Ctrl siRNA transfected SH-SY5Y cells were treated with 0.4mM metformin. Immunofluorescence staining were detected with antibody against α-tubulin(red) and subjected to confocal microscopy analysis. DAPI (blue) was used to stain the nuclei. Scale bars 50μm. Image J software was used to count the cell length. (**E**) Protein levels of FZD3, P-GSK3β, GSK3β, p-tau(ser396) and GAPDH were detected by immunoblotting after 48h treatment of 0.4mM metformin in NEAT1v2 siRNA and Ctrl siRNA transfected SH-SY5Y cells. (mean ± s.d, *P < 0.05, Student 2-tailed *t* test).

Metformin was originally used as an anti-diabetic agent, but recently, it is considered as promising agent for the treatment of AD [35]. Metformin is mainly absorbed by the small intestine, can cross the blood-brain-barrier (BBB) and have a specific effect on the central nervous system (CNS) [36]. Nowadays, clinical and experimental evidence has shown that metformin has beneficial effects on neurodegenerative diseases. To examine whether metformin can prevent tau hyper-phosphorylation caused depolymerization of MTs via FZD3/GSK3β/p-tau pathway, we performed qPCR and immunoblotting to detect the expression change of target genes in metformin treated SH-SY5Y cells. Cell counting kit 8 (CCK8) assay indicated that cell viability is not influenced dramatically only if the metformin concentration is less than 2mM (data not shown). We measured the mRNA level of NEAT1 as well as FZD3 following incubation with increasing concentrations of metformin for 48h, ranged from 0 to 1mM. The expression of NEAT1 and FZD3 rose first and then decreased, and reached to the maximum level after exposure to 0.4mM or 0.6mM metformin, respectively (Figure 5A, 5B). We further detected NEAT1 expression in shNEAT1v2 cells and shCtrl cells both treated with 0 to 1mM metformin and found metformin increase NEAT1 expression in general even though NEAT1was inhibited before (Figure 5C). According to the results, we selected 0.4mM as an optimum concentration for the following experiments.

The neurites damage has already formed and it is difficult to reverse this phenomenon in shNEAT1v2 cells and shCtrl cells by metformin. So we transiently transfected SH-SY5Y cells with NEAT1 siRNA and Ctrl siRNA, simultaneously treated with 0.4mM metformin. Immunofluorescence staining was performed by using anti-α-tubulin antibodies, and quantification demonstrated that the neurites length of NEAT1 siRNA transfected SH-SY5Y cells were significantly prolonged with 0.4mM metformin treatment, indicating that metformin play an essential role in maintaining MTs stability and neurites extension (Figure 5D). Immunoblotting analysis showed increased FZD3 and P-GSK3β expression in NEAT1v2 siRNA transiently transfected SH-SY5Y cells treated with 0.4mM metformin, compared with vehicle treatment. And p-tau protein level was dramatically reduced after exposure to metformin (Figure 5E). Collectively, metformin may rescue the dysregulated FZD3/GSK3β/p-tau pathway and further contributes to MTs stability and neurites extension via increasing NEAT1.

### Metformin decreases tau hyper-phosphorylation in the hippocampi of younger AD mice

**Figure 6:**
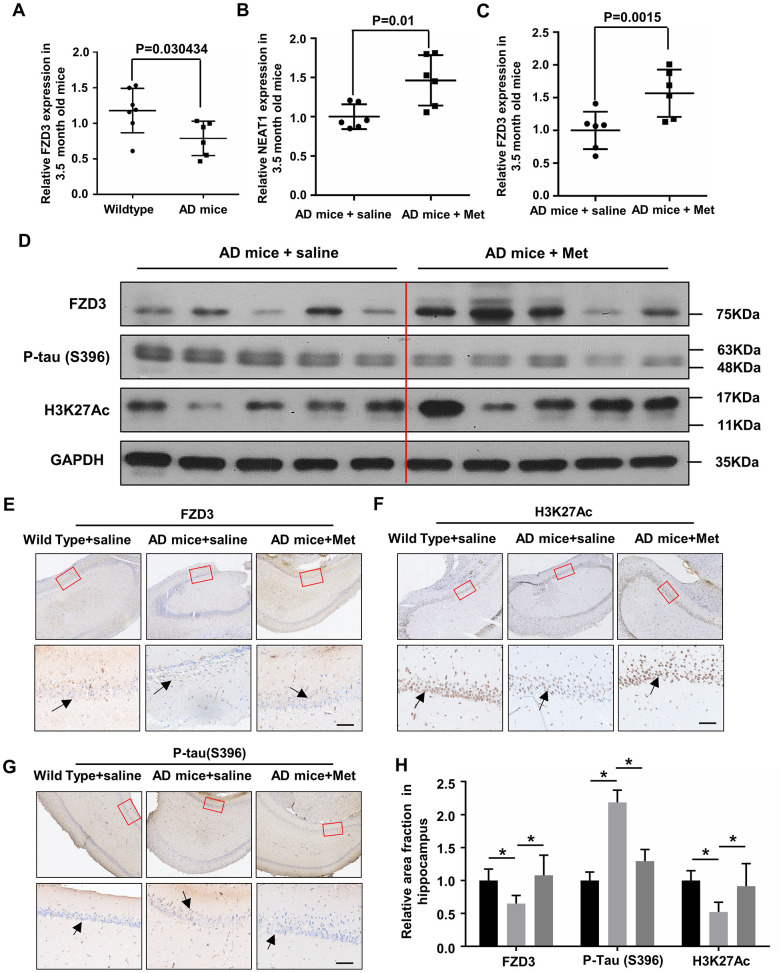
**Metformin abandon tau hyper-phosphorylation in hippocampi of younger APPswe/PS1dE9 double transgenic mice.** (**A**) FZD3 mRNA level in the hippocampi of 3.5-month-old AD mice (n=6) and Wild Type (n=7). (**B**, **C**) Quantitative PCR analysis of NEAT1 and FZD3 expression in the hippocampi of vehicle (n=6) and metformin (MET) administrated (n=6) AD mice for 6 weeks. (**D**) Protein levels of P-tau(S396), FZD3 and H3K27Ac were determined by immunoblotting in the hippocampi of vehicle and metformin intragastric administrated AD mice. (**E**–**G**) The hippocampi of differently administrated AD mice as well as Wild Type were immunohistochemically stained with H3K27Ac, FZD3 and P-tau(S396), Scale bars 50μm. (**H**) Quantification of the relative area fraction occupied by immunostaining of H3K27Ac, FZD3 and P-tau(S396) in CA1 region of hippocampi were analyzed by Image J. (mean ± s.d, *P < 0.05, Student 2-tailed *t* test).

NEAT1 and FZD3 expression were detected in the hippocampi of 3.5-month-old Wild Type and AD mice, and found reduced NEAT1 and FZD3 level in younger AD mice (Figure 1B, 6A). To clarify whether metformin can increase NEAT1 and dephosphorylate tau *in vivo*, we raised 2-month-old AD mice with 6 weeks daily intragastric administration of 200mg/kg metformin as well as saline. Hippocampi from differently administrated AD mice were lysed and analyzed via qPCR and immunoblotting. Increased mRNA levels of NEAT1 and FZD3 were observed in the hippocampi of MET-treated AD mice compared with vehicle-treated AD mice (Figure 6B, 6C). Immunoblotting revealed a significant decrease of Ser396 phosphorylation of tau and an increase of FZD3 and H3K27Ac levels in the hippocampi of MET-treated AD mice (Figure 6D). These results indicated that metformin has an in vivo effect on NEAT1 expression and the phosphorylation of tau. Since CA1 is one of the most affected regions in AD, mainly at early stages [37], we performed immunohistochemical staining and found a decrease of H3K27Ac-positive and FZD3-positive neuronal cells in the CA1 hippocampal area of AD mice compared with Wild Type, but could dramatically increase when administrated with metformin. As for the accumulation of hyper-phosphorylated form of tau, the immunoreactivity was decreased significantly in MET-treated AD mice compared with saline group (Figure 6E–6G). Quantification of the relative area fraction occupied by immunohistochemical staining was analyzed with Image J (Figure 6H). We hypothesized that NEAT1 knockdown in AD mice would lead to hyper-phosphorylated of tau, whereas treatment with metformin would rescue this damage. Indeed, herein, we found that metformin has neuroprotective effect via regulating FZD3/GSK3β/p-tau pathway.

## DISCUSSION

Previous studies have reported that generally increased NEAT1 expression in AD patients and AD mice [19–21, 38], but our results seem to contradict the previous reports. We accidentally found that NEAT1 expression reduced significantly in the early stage of AD and then increased gradually. Excessive accumulation of Aβ and the activation of inflammation are two widely recognized early events in the pathogenesis of AD, which results in the impairments in synaptic functions [39]. NEAT1 and paraspeckle formation are increased in cells exposure to a variety of environmental stressors and implicated in stress response pathways [40]. NEAT1 is a cellular stress sensor and that responds to signals such as inflammation, hot shock, oxidative stress and proteotoxic stress [41, 42]. Accordingly, inflammation, a typical cellular stress, occurs in the early stage of AD, may gradually activate NEAT1 followed, which explains the increased NEAT1 expression in AD patients or APP/PS1 mice.

In this study, we found that depletion of NEAT1 affected MTs stability and inhibited neurites extension in SH-SY5Y cells and murine neurons, and tried to explain this phenomenon. KEGG pathway enrichment analysis revealed that the Wnt signaling pathway is highly enriched in NEAT1- associated genes, and eventually we focused on Wnt ligands receptor FZD3. Emerging evidences suggest that FZD3 involves in the nosogeny of cancer and neuropsychiatric disorders, and especially participates functionally or genetically in schizophrenia [43]. Dysfunction of FZD3 results in neurodevelopmental abnormalities, which accounts for the dysregulated pattern of cortical development and the disruption of major fiber tracts development in the rostral central nervous system (CNS) [44]. Both immunofluorescence staining and immunoblotting analysis revealed that knockdown NEAT1 mediated depolymerization of MTs through FZD3/GSK3β/p-tau pathway, indicating a new mechanism for the aetiology of AD. Furthermore, our previous study revealed that NEAT1 is responsible for amyloid-β deposition as well. We found three genes encoding membrane or membrane-binding proteins (i.e., CAV2, TGFB2 and TGFBR1) regulated by NEAT1, which co-localized with and mediated amyloid-β endocytosis in neuroglial cells [22].

In addition to its function as a fundamental structural component of paraspeckles to regulate gene transcription via nuclear retention of mRNAs, NEAT1 also alter the epigenetic landscape of target gene promoter to favour transcription [22, 32]. Chen et al. reported that knockdown NEAT1 reduced the H3K27me3 in the promoter regions of Wnt signaling regulation factors (Axin2, ICAT, and GSK3β) by interacting with chromosome modification enzyme EZH2 [45]. NEAT1 can associate with chromatin via a specific interaction with histone H3, including the association with active chromatin marks (that is, H3K4me3 and H3K9Ac) [32]. There are several lines of evidence suggesting that NEAT1 might contribute to gene transcription by interacting with chromatin-modifying proteins and/or interacting with histones [32, 46–49]. Previously, we reported that NEAT1 regulates H3K27Ac and H3K27 crotonylation (H3K27Cro) in the promotor region of endocytosis related genes through interaction with P300/CBP complex [22]. And now, ChIP-qPCR and luciferase reporter assay demonstrated that NEAT1 regulate the expression of FZD3 at the transcriptional level and the histone modification H3K27Ac at FZD3 promoter significantly decreased after silencing NEAT1. RIP-qPCR identified that acetyltransferase P300 physically associates with not only NEAT1 but FZD3 promoter region, indicating that NEAT1 influences active transcription mark H3K27Ac on FZD3 promoter by interacting with P300. After that, NEAT1 regulates FZD3/GSK3β/p-tau pathway and may eventually influence AD progression. Our investigation for the first time highlights NEAT1-mediated regulation of FZD3/GSK3β/p-tau pathway.

Accumulating studies indicate that AD and type 2 diabetes are connected at epidemiological, clinical and molecular levels [50, 51]. There is growing evidence for the benefits of metformin to counteract neurodegenerative diseases, such as AD, because of the close association between diabetes and AD. However, the exact mechanism of metformin’s advantageous activity in AD is not fully understood. Researchers reported that both *in vitro* and *in vivo*, metformin could reduce tau phosphorylation in murine neurons via the mTOR/protein phosphatase 2A (PP2A) signaling pathway [25]. Besides, emerging evidences suggest that the potential spectrum of metformin’s beneficial effects also includes anti-inflammatory and anti-oxidative properties [52]. To examine whether metformin could decrease tau phosphorylation via FZD3/GSK3β/p-tau pathway, we performed immunoblotting analysis and found the treatment of metformin upregulated both NEAT1 and FZD3, and eventually reduced p-tau. Furthermore, we identified that metformin could reverse the effect of NEAT1 siRNA on MTs stability and neurites extension in SH-SY5Y cells. Our results thus provide new mechanism that metformin may alleviate AD progression via regulating FZD3/GSK3β/p-tau pathway.

In conclusion, our investigation highlighted a key role of NEAT1 in maintaining MTs stability and neurites extension via regulating FZD3/GSK3β/p-tau pathway, which has significant implications for the aetiology of AD.

## MATERIALS AND METHODS

### Animals

Animals were kept in an environmentally controlled breeding room (temperature: 20 ± 2° C; humidity: 60% ± 5%; 12 h dark/light cycle). The animals were fed standard laboratory chow diets with water ad libitum. The study was performed in strict accordance with the recommendations of the Guide for the Care and Use of Laboratory Animals of the Institutional Animal Care and Use Committee of Tsinghua University. The protocol was approved by the Animal Welfare and Ethics Committee of Tsinghua University, China. C57BL/6 (Wild Type) and APPswe/PS1dE9 double transgenic mice (AD mice) both at the ages of 2 months were obtained from Jackson Laboratory (Bar Harbor, ME, USA) and were housed in individually ventilated cages.

Around 3.5 months old C57BL/6 (n=7) and same age APPswe/PS1dE9 double transgenic mice (n=7) were killed by decapitation to obtain hippocampi tissue and detected mRNA levels of NEAT1 and FZD3. In addition, 2 months old C57BL/6 (n=6) and same age APPswe/PS1dE9 double transgenic mice (n=12) were obtained from the same source and were divided into 3 groups: 1) a sham group (which received daily intragastric administration of saline for 6 weeks, C57BL/6 (n=6).) 2) a control group (which received daily intragastric administration of saline for 6 weeks, AD mice (n=6).) 3) a case group (which received daily intragastric administration of 200mg/kg metformin (Abcam, #ab120847) for 6 weeks, AD mice (n=6).) For Immunostaining, Wild Type and AD mice were killed by decapitation to obtain hippocampi tissue and perfusion were performed with 4% PFA in PBS. To quantify NEAT and FZD3 expression, Wild Type and AD mice were anesthetized with pentobarbital and hippocampal tissue was eluted in RNAiso Plus (Takara, D9108B) followed by RNA extraction.

### Dataset

MRNA expression data sets and the associated clinical information were obtained from GSE84422 (GEO, https://www.ncbi.nlm.nih.gov/gds/) database. GSE84422 is titled as molecular signatures underlying selective regional vulnerability to Alzheimer’s Disease, which includes RNA samples from 19 brain region isolated from the 125 specimens. NEAT1 expression in the hippocampi of 44 AD patients with different braak stage and 11 normal persons was analyzed using nonparametric Kolmogorov-Smirnov test.

### Cell line culture

Human neuroblastoma SH-SY5Y (China Infrastructure of Cell Line Resources) were cultured in DMEM-F12 (Gibco/Invitrogen Ltd, 10565-018) containing 10% fetal bovine serum (Gibco/Invitrogen Ltd, 10099-141C), 10 U/ml penicillin-streptomycin (Gibco/Invitrogen Ltd, 15140-122) in a 5% CO2-humidified incubator at 37° C.

Primary murine neurons were isolated from embryonic E18.5 C57BL/6 mice and approximately 2×10^3^ cells/well were plated on poly-D-lysine-coated glass coverslips (20 μg/ml). The plating medium was DMEM-F12 (Gibco/Invitrogen Ltd, 10565-018) supplemented with 10% horse serum (Gibco/Invitrogen Ltd, 26050-070), 10mM sodium pyruvate (P4562, sigma), 0.5mM glutamine (G6392, sigma) and 1% D-Glucose (G6152, sigma). After 2-4 h, the medium was changed to Neurobasal medium (Gibco/Invitrogen Ltd, 21103-049) supplemented with 2% B27(Gibco/Invitrogen Ltd, 17504-001), 1% N-2 Supplement (Gibco/Invitrogen Ltd, A13707-01) and 1% L-Glutamine (Gibco/Invitrogen Ltd, A2916801)). Besides, 5ug/ml AraC (C6645, sigma) was added to neuronal growth medium after 72h to inhibit glial growth. Lentivirus transfections were conducted as follows.

### Cell transfections

All the synthetic lentivirus-based shRNAs (shNEAT1v2, shNEAT1-Mus and shCtrl) and siRNAs (NEAT1v2#1 siRNA, NEAT1(v1+v2) #2 siRNA, FZD3#1 siRNA, FZD3#2 siRNA, FZD3#3 siRNA and Ctrl siRNA) were purchased from Shanghai GenePharma Co., Ltd. All the siRNAs were transfected with lipofectamine™ 2000 (Invitrogen, 11668-019) according to the manufacturer’s protocol, and shRNAs were co-transfected with polybrene (GenePharma Co. (Shanghai, China)). The siRNA sequences were shown in Table 3.

**Table 3:** List of siRNAs sequence.

**siRNA**	**Sequence**
Ctrl siRNA	UUCUCCGAACGUGUCACGU
NEAT1v2#1 siRNA	CAAACUCUGUACCCAUUAA
NEAT1(v1+v2)#2 siRNA	CCUCUACUACAAGCACCUGAA
FZD3#1 siRNA	GGAGAACCAAGAUAAAUUA
FZD3#2 siRNA	AUACCUGAUGGCUCUCAUA
FZD3#3 siRNA	AUACUCCUAUCAUAAGAAA

### Construction of stable cell line

Human neuroblastoma SH-SY5Y cells were cultivated in 6-well plate (1x10^4^/well). When the cell density reached 40-50% confluence, the Lentivirus based shRNAs were used for cell transfection to generate stable NEAT1-depletion monoclonal cell line (shNEAT1v2 cells and shCtrl cells). Puromycin (A1113803, Invitrogen; Thermo Fisher Scientific, Lnc) selection (10ug/ml) started 24h after transfection. The medium with 10 μg/ml puromycin was changed every 2-3 days. Following 2-4 weeks, isolated colonies were selected and grown for later assays.

### Reverse transcription and quantitative PCR

Reverse transcription was performed using ReverTra Ace® qPCR RT Master Mix with gDNA remover (TOYOBO, FSQ-301) according to the manufacturer’s protocol. The resultant cDNA was measured by quantitative PCR using following system: 4μl of RNase-free H2O, 0.5 μl of forward primer (1 μM), 0.5ul of reverse primer (1 μM), 1 μl of cDNA (50 ng) template, and 5 μl of SYBR Green PCR Master Mix (TOYOBO, QPK-201) at 95° C for 30 s, followed by 40 cycles of 95° C for 15 s, 60° C for 15 s and 70° C for 15 s. All mRNA levels were normalized to beta-actin. The primers were shown in Table 4.

**Table 4:** List of primers used for RNA analyses.

**Primers pairs**	**Sence sequence**	**Anti-sence sequence**
hNEAT1v2	ACATTGTACACAGCGAGGCA	CATTTGCCTTTGGGGTCAGC
mNEAT1v2	CTTGCCACACCTTGTCTTGC	TAGCTGGTGCATCCTGTGTG
hFZD3	GTTCATGGGGCATATAGGTGG	GCTGCTGTCTGTTGGTCATAA
mFZD3	GTTACCACTTGGAGAGGCCC	AACCTGGCGCAGTAACATGA
hACTB	GACGTGGACATCCGCAAAG	CTGGAAGGTGGACAGCGAGG
mACTB	TACCCAGGCATTGCTGACA	GCAGCTCAGTAACAGTCCG

### Immunoblotting

The antibodies used for immunoblotting included an anti-GSK3β antibody (cell signaling, #12456), an anti-Phospho-GSK3β(ser9) antibody (cell signaling, #9322), an anti-FZD3 antibody (Abcam, ab75233), an anti-phospho-Tau (S396) antibody (Abcam, ab109390), an anti-Phospho-Tau (Ser400/Thr403/Ser404) antibody (Cell signaling, #11837), an anti-acetyl-Tubulin antibody (Sigma, T6793) and an anti- GAPDH antibody (Proteintech,10494-1-AP) was analyzed by western blot. Protein sample was lysed in ice-cold whole cell extract buffer B (50 mM TRIS-HCl, pH 8.0, 4M urea and 1% Triton X-100), followed by being heated at 100° C for 10 min with 5× loading buffer. Then, equal amount of protein sample was separated by SDS-PAGE and transferred onto PVDF membranes (Millipore, Immobilon-NC). Membranes were blocked for 1h at room temperature with 5% non-fat milk and successively incubated overnight at 4° C with primary antibody and 2h room temperature for secondary antibody. After that, ECL Blotting Detection Reagents were used to visualize protein bands.

### Immunofluorescence

Antibodies against α-tubulin (Sigma, T6074) and the secondary antibodies Alexa Fluor 594 (Life Technologies Corp.) were employed in immunofluorescence staining. Microscopic analysis that clearly reflecting changes of axonal length were captured using an Olympus FV1000 confocal laser microscope.

### Luciferase assay

The dual-luciferase promoter assay system was generated by inserting sequences from -500bp to +500bp relative to the transcription start sites (TSS) of FZD3. The inserted reporters were obtained from Shanghai GenePharma Co. Ltd. Luciferase activities were assayed using a Dual-Luciferase Reporter System (Promega, E1960).

### ChIP assay

ChIP assays were performed as described previously [53]. Briefly, the cells were digested with ChIP lysis buffer (50 mM Tris-HCL PH=8.0, 5 mM EDTA, 0.1% deoxycholate, 1% Triton X-100, 150 mM NACL in 1* PIC (protease inhibitor)), And were crosslinked with 1% formaldehyde and sonicated for 180s (10s on and 10s off) on ice shear the DNA to an average fragment size of 200-1000bp. The 500ul of sonicated chromatin was purified by centrifugation, and then, the supernatants were incubated with the ChIP grade antibody against 2-5ug anti-KAT3B/P300 (Abcam, ab54984), anti-Histone H3 (acetyl K27) (Abcam, ab4729) and 100ul Dynabeads^TM^ protein G (Invitrogen, 10004D, USA). Finally, chromatin DNA was subjected to Quantitative PCR and all primers for ChIP-qPCR are listed in Table 1.

### RNA immunoprecipitation and RIP-qPCR assays

The NEAT1 deficient cell lines were lysed in polysome lysis buffer (10mM KCl, 5mM MgCl2, 10mM HEPES PH7.0, 0.5% NP-40, 1mM DTT, 100U/ml RRI, 20ul/ml PIC, 2mM vanadyl ribonucleotide complex solution.). Cell lysates were incubated with TE5.0 buffer (10mM Tris-HCl, 10mM EDTA, PH=5.0) containing magnetic beads (Dynabeads^TM^ protein G, Invitrogen, 10004D, USA) conjugated with IP grade antibody anti-KAT3B/P300 (Abcam, ab54984) and the negative control (normal mouse IgG; ab190475, abcam). Purified RNA was obtained, and qRT-PCR was performed with the NEAT1 primers to demonstrate the presence of the binding targets. The primer used in qRT-PCR were shown in Table 2.

### CCK8 assay

Approximately 5x10^3^ cells per well were plated in a 96-well culture plate and incubated 24 h, followed by treating different concentrations of metformin for 48 h. Then, 10 μl CCK8 reagent (MedChem Express, HY-K0301-500T, China) were added to each well and maintained for 4h at 37° C After that, the absorbance at 450nm was detected by using a microplate reader.

### Immunohistochemistry

The hippocampi of mice were isolated and fixed in 4% paraformaldehyde (PFA) and then placed in 30% sucrose in PBS for 1 day. Paraffin-embedded tissue sections (10um thickness) were treated in 0.01M PBS containing 3% hydrogen peroxide (H_2_O_2_) for 10 min. Then blocked in 3% BSA and were incubated in following antibodies: an anti-phospho-Tau (S396) antibody (Abcam, ab109390), an anti-FZD3 antibody (Abcam, ab75233) and an anti-acetyllysine antibody (Cat#PTM-102, clone Kac-11). Specimens were visualized under an inverted phase contract fluorescent microscope [54].

### Statistical analysis

All assays were repeated at least three times and data are shown as means ±SD. P values were determined by comparing the data from treated and control cells. Data were evaluated with two-tailed t-test. Differences were considered significant with a value of *P < 0.05, **P < 0.01.
